# From an unusual organotin(IV) coordination compound to the first ionic organic–inorganic mixed-valent tin(IV)–tin(II) compound

**DOI:** 10.1107/S2056989026006596

**Published:** 2026-06-26

**Authors:** Daniel Schwarte, Swantje Warf, Martin Reichelt, Hans Reuter

**Affiliations:** a Chemistry, Osnabrück University, Barbarastr. 7, 49069 Osnabrück, Germany; University of Hyogo, Japan

**Keywords:** tetrel bonds, mixed-valent, coordination compounds, crystal structure, tin

## Abstract

In the search for complexes of the monoorganotin(IV) triiodides, *R*SnI_3_, with Lewis bases, LB, a 1:3 complex was was obtained for the first time in the case of iso­propyl­tin(IV) triiodide, ^*i*^PrSnI_3_, and LB = pyridine-*N*-oxide, PyNO. The compound, di­iodido­(isoprop­yl)tris­(pyridine *N*-oxide)tin(III) iodide, [Sn(C_3_H_7_)I_2_(C_5_H_5_NO)_3_]I, has an ionic structure and consists of a previously unknown [^*i*^PrSn(pyNO)_3_]^+^ and an isolated I^−^ ion. The novel cation exhibits a facial arrangement of the PyNO mol­ecules and a *cis* arrangement of the iodine atoms. In CDCl_3_, over the course of several weeks, this compound gives rise to a new, second compound, also of ionic structure, containing an tri­iodido­stannate(II) ion, [SnI_3_]^−^, and a solvent mol­ecule in addition to the already known cation, namely, di­iodido­(isoprop­yl)tris­(pyridine *N*-oxide)tin(IV) tri­iodido­stannate(II) deutero­chloro­form monosolvate, [Sn(C_3_H_7_)I_2_(C_5_H_5_NO)_3_][SnI_3_]·CDCl_3_. The anion exhibits a trigonal–pyramidal structure in order to achieve a stable electron octet at the divalent tin atom but is associated in the crystal *via* tetrel bonds into one-dimensional chains in which the tin atoms exhibit a 3_3_-aaa coordination mode.

## Abstract 2

The previously unknown 1:3 complex of iso­propyl­stannate(IV) iodide, ^*i*^PrSnI_3_, and pyridine *N*-oxide, PyNO, partially decomposes upon prolonged storage in deutero­chloro­form, forming a novel mixed-valent organic–inorganic tin(IV) and tin(II) compound. Both compounds exhibit an ionic structure and contain an octa­hedral [^*i*^PrSnI_2_(pyNO)_3_] ^+^ ion, in which the three Lewis base mol­ecules are arranged in a *facial* configuration and the two iodine atoms are in a *cis* position, whilst charge balancing is ensured in the first compound by an isolated iodide ion and in the second by a tri­iodido­stannate(II) ion, [SnI_3_]^−^, which is linked *via* tetrel bonds to one-dimensional chains.

## 1. Introduction

The excellent coordination behaviour of organotin(IV) halides, *R*_4–*n*_SnHal_*n*_ with Hal = Cl, Br, I, and *n* = 1, 2, 3 has been known for over 100 years (Krause & von Grosse, 1937 ▸). Nevertheless, it is surprising how little is still known today about the structures of complexes of monoorganotin(IV) trihalides, *R*SnHal_3_, particularly those containing the halogens bromine and iodine. In the case where Hal = I, only three crystal structures are described in the literature, all with *R* = ethyl and two Lewis base mol­ecules LB: EtSnI_3_(Ph_2_SO)_2_ (Jatsenko *et al.*, 1985 ▸), EtSnI_3_(Ph_3_PO)_2_ (Tursina *et al.*, 1986 ▸) and EtSnI_3_(HMPTA)_2_ (Aslanov *et al.*, 1985 ▸). In all three compounds, the tin atoms are coordinated in a distorted octa­hedral arrangement, but with different stereochemistry: the first compound exhibits a *mer*–*cis* configuration, the second a *mer*–*trans* configuration and the third a *fac*–*cis* configuration with respect to the three iodine atoms and the two Lewis base mol­ecules. Thus, these complexes simultaneously represent all three possible arrangements of ligands in octa­hedral complexes of the composition *R*SnHal_3_LB_2_.

Here we report on our search for suitable complexes containing pyridine-*N*-oxide, PyNO, as a Lewis base. In the case of iso­propyl­tin(IV) triiodide, ^*i*^PrSnI_3_, we were able to isolate a compound of composition ^*i*^PrSnI_3_·3PyNO, **1**, in which, for the first time, three Lewis base mol­ecules are incorporated. Moreover, this compound decomposed partially giving rise to single crystals of a mixed-valent tin(IV)-tin(II) compound of overall composition ^*i*^PrSnI_3_·SnI_2_·3PyNO·CDCl_3_, **2**.

## 2. Results and discussion

Single crystals of compound **1** were first synthesized on a Petri dish by adding iso­propyl­tin(IV) triiodide to an excess of pyridine-*N*-oxide using chloro­form as the solvent. As the elemental analysis carried out indicated a significantly higher C and H content than would be expected for a 1:2 complex a single crystal structure X-ray analysis was performed with a needle-like fragment of a larger yellow bloc, confirming the 1:3-composition according to its constitution of [^*i*^PrSn^IV^I_2_(pyNO)_3_]I. Based on this stoichiometry, the compound was then synthesized on a micro-scale and fully characterized by ^1^H and ^13^C NMR spectroscopy and elemental analysis.

During the spectroscopic characterization process, the NMR tube remained unemptied for several weeks due to bottlenecks in the waste disposal process. Thereafter, several crystals were found on the inner wall, their shape clearly differing from that of the crystals originally placed there. The subsequent X-ray structure analysis revealed the unexpected formation of the ionic organic-inorganic mixed-valent tin(IV)-tin(II) compound **2** with the constitution of [^*i*^PrSn^IV^I_2_(pyNO)_3_][Sn^II^I_3_]·CDCl_3_.
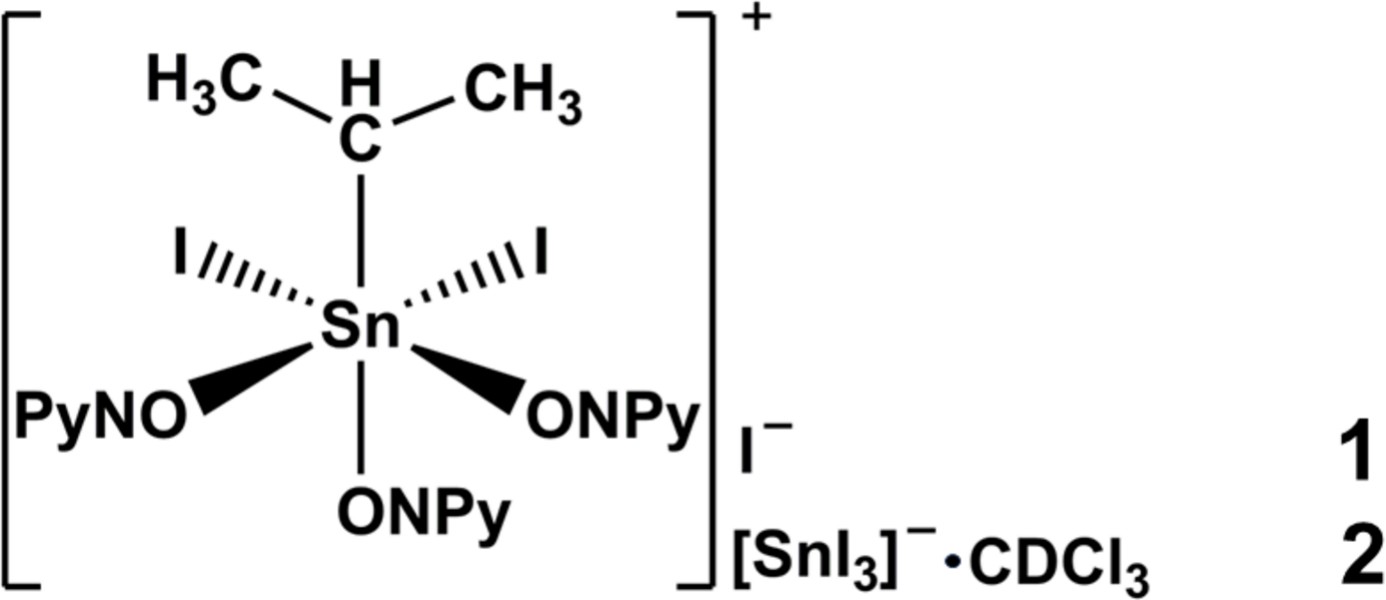


**Figure 1:**
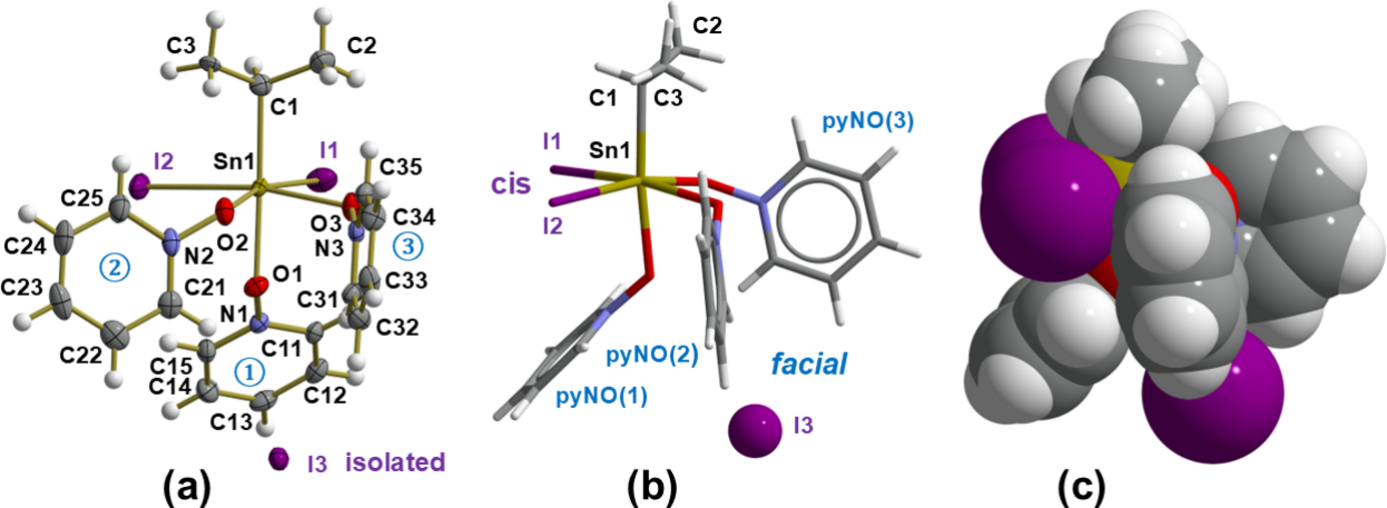
Different representations of the asymmetric unit of **1** reflecting the main component of the disordered iodine atoms and one position of the disordered isopropyl group: (**a**) ball-and-stick model with atom and pyridine *N*-oxide (in circles) numbering. With the exception of the hydrogen atoms, which are shown as spheres of arbitrary radius, all other atoms are drawn as anisotropic displacement ellipsoids at the 60% probability level, (**b**) ball-and-stick model illustrating the stereochemical descriptors of the cation, and (**c**) space-filling model visualizing the shape of the cation; colour code and van der Waals radii used: Sn = bronze, 2.17 Å; I = violet, 1.98 Å; C = dark grey, 1.70 Å; H = white, 1.20 Å; O = red, 1.52 Å; N = light blue, 1.55 Å.

Both compounds are ionic in nature and contain a previously unknown [*R*Sn^IV^Hal_2_(LB)_3_]^+^ ion with *R* = ^*i*^Pr, Hal = I, and LB = PyNO. In this cation (Fig. 1 ▸), the tin atom has a distorted octa­hedral coordination, with the three PyNO mol­ecules adopting a *fac* configuration and the iodine atoms being in a *cis* position relative to one another which results in the organic moiety being in a *trans* position to one of the three PyNO mol­ecules.

Some conformational flexibility of this cation is indicated in the case of the isopropyl group which is statistically disordered over two sets of sites with the same degree of occupancy as well as in a slight disorder (∼97:3) of the iodine atoms in **1**, and in some different orientation of the PyNO mol­ecules in **1** and **2**. Some characteristic structural features of the cation are summarised in Table 1 ▸. More remarkable, however, are the unusual long tin–carbon distances. In comparable but neutral compounds such as ^*i*^PrSnCl_3_(LB)_2_, the tin–carbon bond lengths are also relatively long [2.169 (5)/2.171 (4) Å (Reuter *et al.*, 1992 ▸), LB = DMF; 2.148 (6)–2.177 (3) Å (Kastner *et al.*, 1999 ▸), LB = DMSO], but significantly shorter than in the present case [2.219 (5) Å, **1**; 2.229 (6) Å, **2]**. However, it appears that long tin–carbon bond lengths are a characteristic feature of monoorganotin(IV) iodine compounds as comparable or longer values are found in the complexes *mer*,*cis*-EtSnI_3_·2Ph_2_SO [*d*(Sn—C) = 2.22 (1) Å; Yatsenko *et al.*, 1985 ▸], *fac*,*cis*-EtSnI_3_·2HMPTA [*d*(Sn—C) = 2.25 (3); Aslanov *et al.*, 1985 ▸] and *mer*,*trans*-EtSnI_3_·2Ph_3_PO [*d*(Sn—C) = 2.25 (1) Å; Tursina *et al.*, 1986 ▸] at room temperature.

The Sn—I bond lengths (main component of **1**) ranging from 2.7886 (4) to 2.8468 (4) Å are, on average, shorter than those in the neutral *R*SnI_3_·2LB complexes mentioned above for which values between 2.821 (1) and 2.949 (3) Å are found at room temperature. Significantly shorter [2.634 (3); 2.715 (2) Å] Sn—I distances are found there only in the case of iodine atoms that are in a *trans* position relative to the organic moiety. This bond shortening is usually referred to as the *trans strengthening* (Jatsenko *et al.*, 1985 ▸).

Pyridine *N*-oxide complexes of monoorganotin(IV) tri­halides have not yet been described in the literature. With regard to the Sn—O bond lengths, there is no consistent pattern in the two cations described here. In compound **1**, the Sn—O bond in the *trans* position relative to the organic moiety is significantly shorter [2.131 (2) Å] than the two Sn—O bonds in the *cis* positions [2.181 (4), 2.182 (4) Å]. In compound **2**, one Sn—O bond in the *cis* position is of similar [2.184 (4) Å] length to that in compound **1**, but the second Sn—O bond in the *cis* position [2.161 (3) Å] is almost as long as the one in the *trans* position [2.165 (2) Å], with both being significantly longer than the *cis* bonds in compound **1**.

The structural changes in the pyridine *N*-oxide mol­ecules resulting from their inter­action with the tin atoms are most pronounced in the N—O bond lengths, which are slightly longer than in the free mol­ecule [1.306 (2) Å, *T* = 173 K; Shishkin *et al.* 2013 ▸] whereby the bond elongation is all the more pronounced [**1**: *d*(N—O)_*trans*_ = 1.352 (4) Å, *d*(N—O)_*cis*_ = 1.347 (4)/1.249 (4) Å; **2**: *d*(N—O)_*trans*_ = 1.353 (6), *d*(N—O)_*cis*_ = 1.247 (5)/1.352 (6) Å] the stronger the mol­ecule is bound to the tin atom. The associated Sn—O—N bond angles are 120.4 (2)–125.3 (2)° in **1** and 123.1 (3)–127.3 (3)° in **2**.

**Figure 2:**
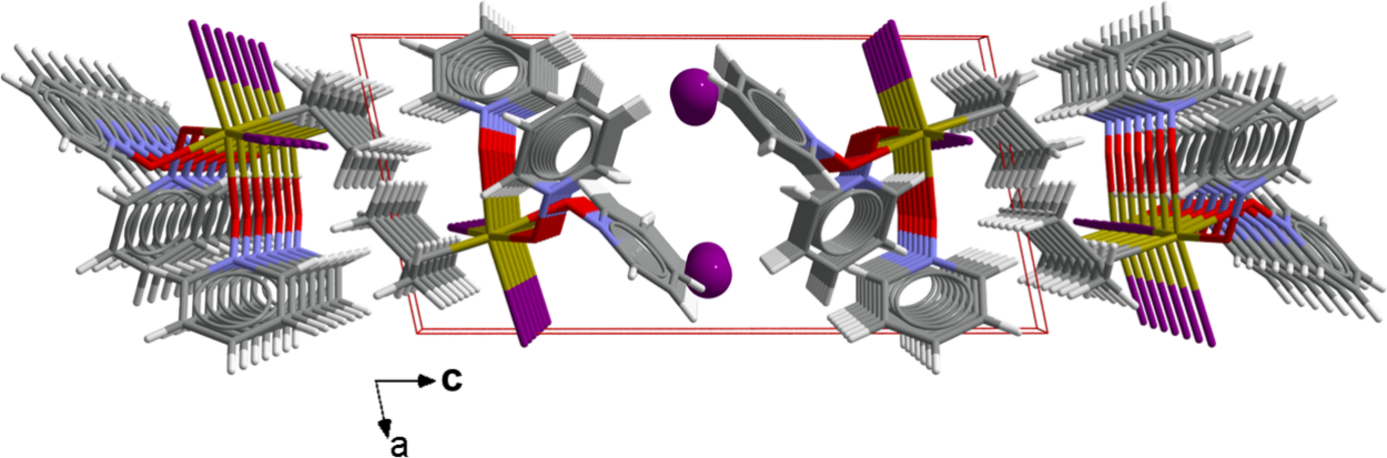
Perspective view into the crystal structure of **1** looking down the *b* axis; cations are drawn as ball-and-stick models, the isolated iodine atoms as spheres of arbitrary radii, colours as shown in the previous illustration.

In **1**, the isolated iodine anions are arranged in layers perpendicular to the *c* axis (Fig. 2 ▸). The individual layers are separated by bilayers of cations in which the isopropyl groups face inwards and the pyridine *N*-oxide mol­ecules face outwards. In this arrangement, the inter­actions between the individual building units are limited to van der Waals contacts.

**Figure 3:**
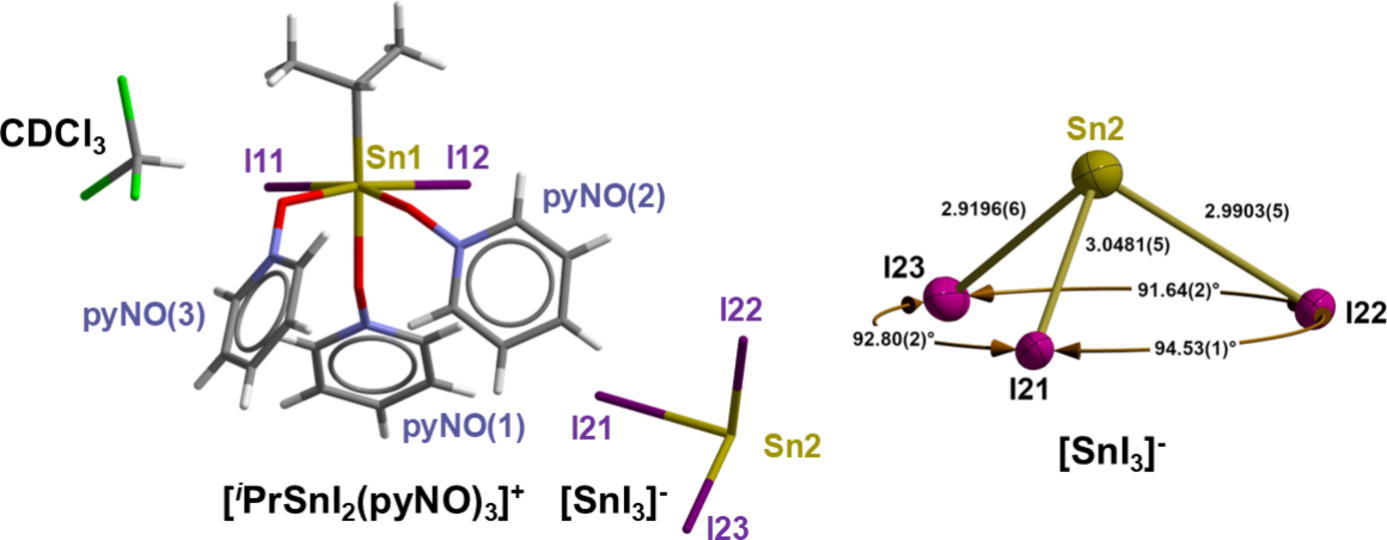
(**left**) Ball-and-stick model of the asymmetric unit of **2** with numbering of selected atoms and numbers of the pyridine-*N*-oxide ligands, numbering of all other atoms according to the numbering scheme of **1**, (**right**) ball-and-stick model of the trigonal–pyramidal [SnI**_3_**]^−^ ion with atom numbering and bond lengths (Å) and bond angles (°), all atoms are drawn as thermal displacement ellipsoids at the 60% probability level.

The three building units in the asymmetric unit of **2** are shown in Fig. 3 ▸*a*. Unlike in **1**, there is no disorder in the cation. The [SnI_3_]^−^ ion (Fig. 3 ▸*b*) has a pyramidal shape with three iodine atoms at the base and the tin atom at the apex. In this species, the tin atom achieves a stable octet of electrons *via* its spherical, non-bonding 5*s* electron pair and the six electrons in the 2*e*–2*c* bonds with the iodine atoms, in which its three orthogonal 5*p* orbitals are involved. Accordingly, the bond angles between the iodine atoms vary between 91.64 (2) and 94.53 (1)°. What is striking, however, are the varying tin–iodine distances, which range from 2.9196 (6) to 3.0481 (5) Å.

The existence of the [Sn^II^I_3_]^−^ ion naturally raises the question of how it is formed. In most cases of incidentally discovered mixed-valent tin(II)–tin(IV) compounds, their formation is based on the partial oxidation of a tin(II) species to tetra­valent tin. In the present case, however, it is evidently a matter of the partial reduction of a tin(IV) species to divalent tin. It remains unclear to what extent the cleavage of the tin–carbon bond or the oxidation of iodide ions to elemental iodine play a role in this process, or whether both reactions are involved, as neither their reaction products nor a violet colour in the reaction solution were observed.

**Figure 4:**
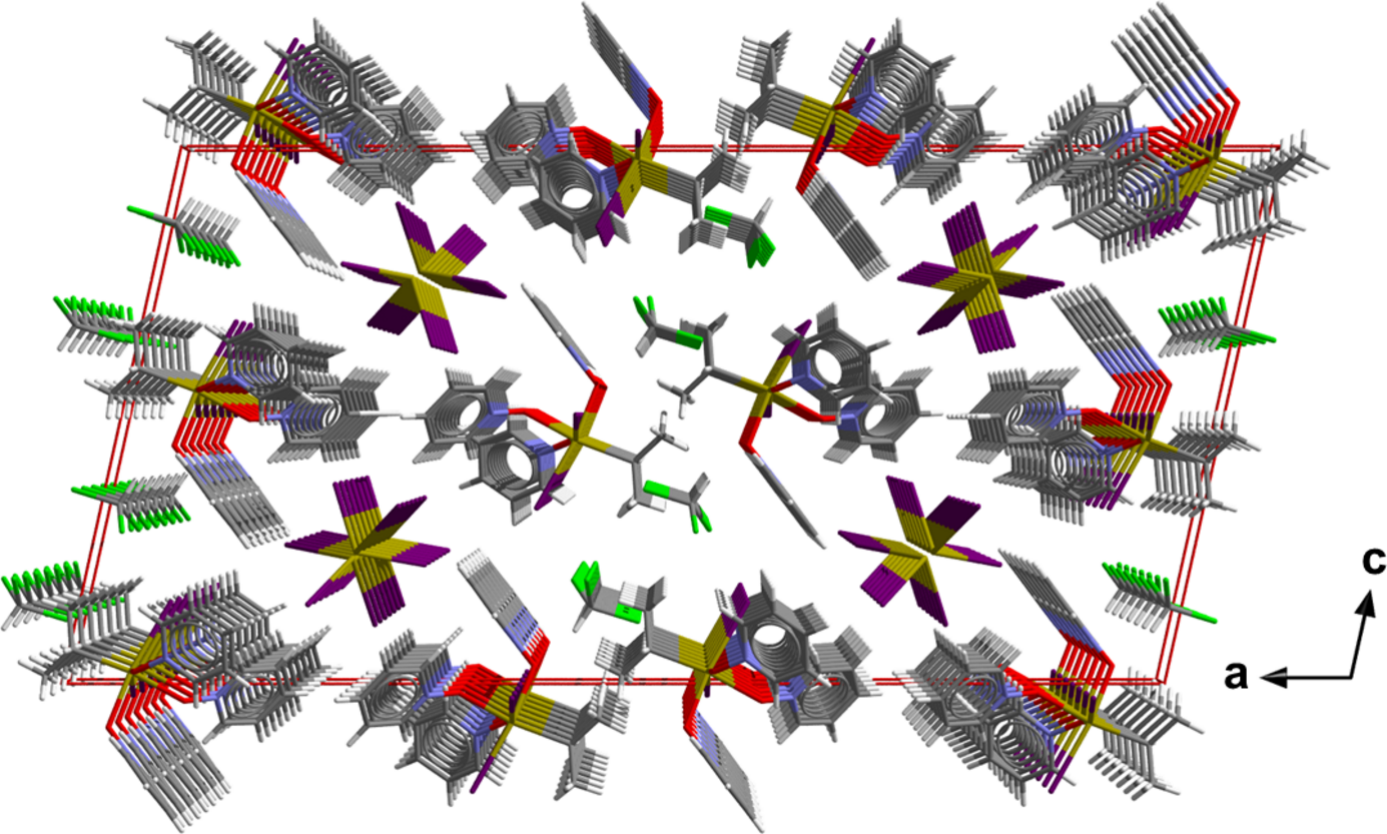
Perspective view into the crystal structure of **2** looking down the *b* axis, all components are drawn as ball-and-stick model using the previous colour code with the addition for Cl = green.

**Figure 5:**
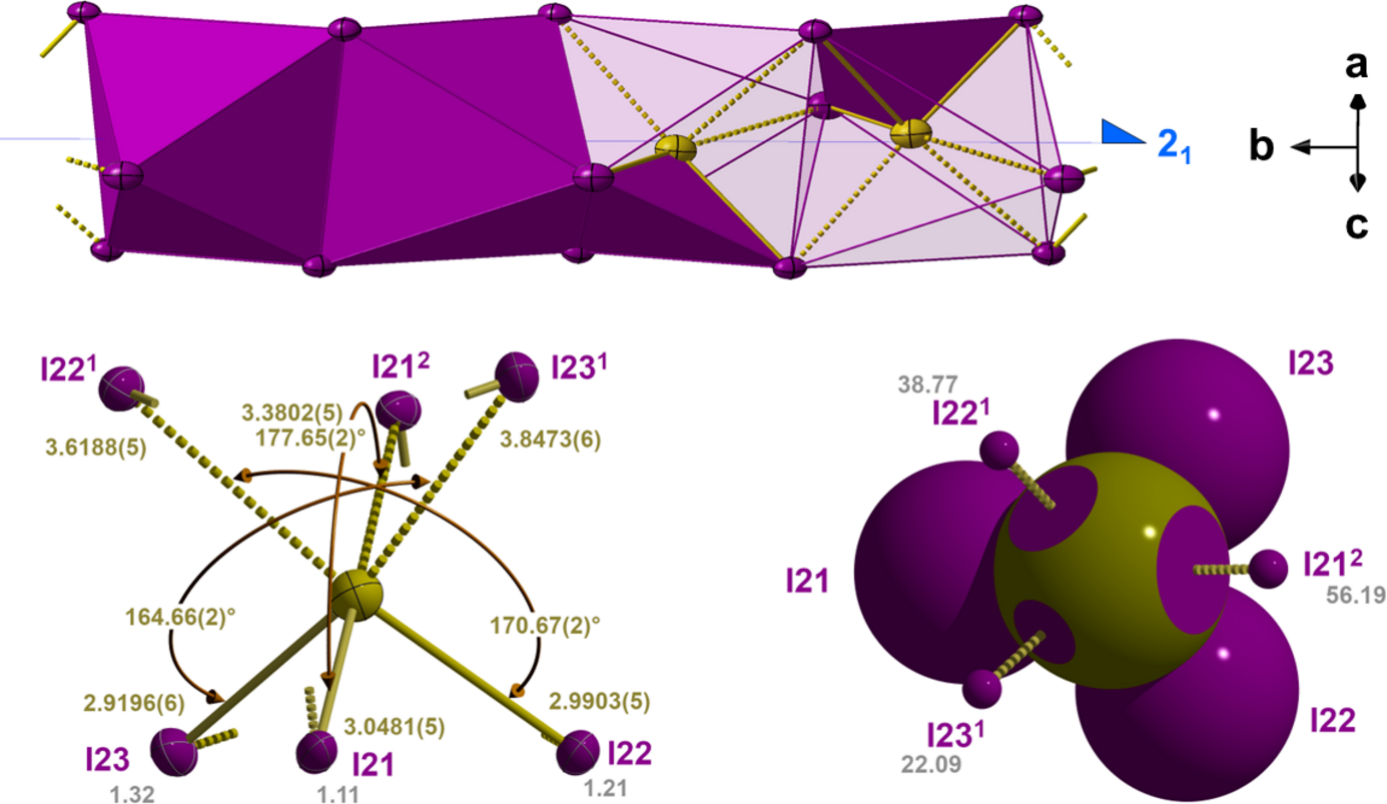
Different representations of the tetrel bonds linking the [SnI_3_]^−^ ion into linear chains in direction of the twofold rotation axis. (**above**) side-view on a chain as polyhedron model with resulting octa­hedra (left) and constituting trigonal-pyramids (right), all atoms are drawn as thermal displacement ellipsoids at the 60% probability level and the tetrel bonds as dashed sticks, (**below**, **left**) geometric parameters [Å,°] characterizing the tetrel bonds with asymmetry parameters Q (grey), and (**below**, **right**) space-filling model visualizing the inter-penetration of the van der Waals radii of tin and iodine as result of the tetrel bond formation, inter-penetration indices *p* (grey), colour code and van der Waals radii as previously.

A look inside the crystal structure of **2** (Fig. 4 ▸) reveals that the cations are arranged in a similar way to those in the parent compound **1**, namely through the inter­action of their isopropyl groups. The [SnI_3_]^−^ ions (Fig. 5 ▸) are arranged in rows along the *b*-axis direction related to each other *via* the twofold screw axis giving rise to some additional, long-range Sn⋯I distances, expanding the original coordination numbers of the tin atoms from three, trigonal-pyramidal, to six in a distorted octa­hedral fashion. Even though long [3.3802 (5)–3.8473 (6) Å], the resulting tin–iodine distances are shorter than the sum (4.15 Å) of the van der Waals radii (Mantina *et al.*, 2009 ▸) of tin (2.17 Å) and iodine (1.98 Å), leading to a considerable inter-penetration of their van der Waals spheres qu­anti­fied by high inter-penetration indices *p* (Echeverría & Alvarez, 2023 ▸).

Such additional weak inter­actions are typical of many tin(II) compounds and are always found on the opposite side to the strong, regular bonds *via* which the tin(II) atom achieves the electron octet. They belong to the tetrel bonds (Bauzá *et al.* 2019 ▸; Brammer *et al.* 2023 ▸) or more specifically to the stannic bonds (Reuter, 2025 ▸) and are usually explained by 3*c*–4*e* bonds between the empty orthogonal 5*p* orbitals of the tin(II) atom and the double-occupied *p*-orbitals of two *trans*-configurated electron-donor atoms *X*. Only rarely are the two donor atoms equidistant from the central tin atom and is the 3*c*–4*e* bond symmetric (s); an example of this can be found in one of the two tin atoms in tin diiodide, SnI_2_ (Howie *et al.* 1972 ▸). Much more frequently, the two donor atoms are at different distances from the tin atom, as shown here, making the 3*c*–4*e* bond asymmetrical (a). Qu­anti­tatively, the degree of asymmetry in such a *trans*-figured *X*—Sn⋯*Y* arrangement can be determined by the quotient *Q* = *d*(Sn⋯Y)_long_/*d*(Sn—*X*)_short_ (Schröder *et al.*, 2024 ▸). In the present case these values are 1.11, 1.21, and 1.32, indicating a strong asymmetry. Based on these observations, the extended coordination of the tin(II) atoms of the [SnI_3_]^−^ ions should be described as 3_3_-aaa coordination mode (Schröder *et al.*, 2024 ▸), which is more precise than the term ‘distorted octa­hedral’.

## 3. Experimental

### 3.1. Synthesis and crystallization

^*i*^PrSnI_3_: under stirring, a solution of 6.75 g (25 mmol) of iso­propyl­tin(IV) trichloride, ^*i*^PrSnCl_3_, in acetone (50 ml) was added to a solution of 11.25 g (75 mmol) of sodium iodide in acetone (120 ml). After stirring for 1 h, the solid formed was filtered off and the solution was evaporated down in a rotary evaporator. The remaining residue was distilled by fractional distillation (b.p.: 367–368 K/20 mbar, light yellow, oily liquid), yield: 8.71 g (16.1 mmol, 64%).

^1^H NMR (250 MHz, CDCl_3_): δ, *^n^J*(^119/117^Sn—^1^H) (ppm, Hz) 1.21, 216.7/207.4 (*d*, –C*H*–, 1H); 2.70 (*sep*, –CH_3_, 6H); ^13^C NMR (250 MHz, CDCl_3_): δ, *^n^J*(^119/117^Sn—^13^C) (ppm, Hz) 20.18, 387.3/370.2 (–*C*H_3_), 37.65, 500.3/478.0 (–*C*H–); analysis: calculated for C_3_H_7_I_3_Sn (542.51): C 6.64, H 1.30; found: C 6.69, H 1.35%.

^*i*^PrSnI_3_·3PyNO, **1**: in a beaker, 0.54 g (1 mmol) of iso­propyl­tin(IV) triiodide, ^*i*^PrSnI_3_, and 0.28 g (3 mmol) of pyridine *N*-oxide (Sigma-Aldrich) were dissolved in 20 ml of chloro­form. Upon slow evaporation of the solvent in air, the complex crystallized as yellow, translucent crystals, which were dried between two filter papers, yield: 0.47 g (0.57 mmol, 85%).

^1^H NMR (250 MHz, CDCl_3_): δ, *^n^J*(^119/117^Sn—^1^H) (ppm, Hz) 1.21, 281.1/267.9 (*d*, C*H*, 1H); 2.70 (*septet*, C*H*_3_, 6H) 7.49–7.63 (multiplet, *meta*-, *para*-*H*_pyNO_, 9H), 8.52 (*d*, *ortho*-*H*_pyNO_, 6H); ^13^C NMR (250 MHz, CDCl_3_): δ, *^n^J*(^119/117^Sn—^13^C) (ppm, Hz) 21.54, 46.2 (–*C*H_3_), 48.79 (–*C*H–), 126.39 (*meta*-*C*_pyNO_), 130.91 *para*-*C*_pyNO_), 140.66 (*ortho*-*C*_pyNO_); analysis: calculated for C_18_H_22_I_3_N_3_O_3_Sn_2_ (827.77): C 26.12, H 2.68, N 5.08; found: C 26.43, H 2.72, N 5.13%.

Single crystals of [^*i*^PrSn^IV^I_2_(pyNO)_3_][Sn^II^I_3_] · CDCl_3_, **2**, were obtained after the NMR tube had been left standing for some time.

### 3.2. Refinement

Crystal data, data collection and structure refinement details are summarized in Table 2 ▸. Hydrogen atoms were refined with calculated positions (–CH– = 1.00 Å, –CH_3_ = 0.98 Å, –CH_pyNO_ = 0.95 Å, AFIX) and isotropic displacement parameters *U*_iso_(H) = *P* × *U*_eq_(C) with *P* = 1.2 for all hydrogen atoms without those of the methyl groups (*P* = 1.5).

Disorder of the isopropyl group in the crystal structure of **1** has been modeled *via* tin–carbon and carbon–carbon constrains (DFIX) and common anisotropic temperature factors while occupation factors were fixed to 0.5. In the case of the disordered iodine atoms in the cation of **1**, the occupancy factors (0.967/0.033 for I1, 0.969/0.031 for I2) and positions were freely refined with the anisotropic displacement parameters of the main components.

## Supplementary Material

Crystal structure: contains datablock(s) 1, 2. DOI: 10.1107/S2056989026006596/ox2024sup1.cif

Structure factors: contains datablock(s) 1. DOI: 10.1107/S2056989026006596/ox20241sup2.hkl

Structure factors: contains datablock(s) 2. DOI: 10.1107/S2056989026006596/ox20242sup3.hkl

CCDC references: 2564137, 2564136

Additional supporting information:  crystallographic information; 3D view; checkCIF report

## Figures and Tables

**Table 1:** Selected atom distances and angles (Å, °) in the [^*i*^PrSnI_2_(pyNO)_3_] ^+^ ion of **1** and **2**

	**1**	**2**
*d*(Sn—C)	2.219 (5)^*a*^	2.229 (6)
*d*(Sn—I)	2.7886 (4)	2.8145 (5)
	2.8468 (4)	2.8206 (6)
*d*(Sn—O)_*trans*_	2.132 (3)	2.169 (3)
*d*(Sn—O)_*cis*_	2.181 (3)	2.185 (4)
	2.184 (3)	2.163 (4)
(C—Sn—O)_*trans*_	173.3 (1)/155.2 (3)	165.0 (2)°
(I—Sn—O)_*trans*_	166.9 (1)	169.4 (1)
	169.2 (1)	161.9 (1)

Note: (*a*) Refined value for both positions of the disordered isopropyl group.

**Table 2:** Experimental details

	**1**	**2**
Crystal data		
Chemical formula	[Sn(C_3_H_7_)I_2_(C_5_H_5_NO)_3_]I	[Sn(C_3_H_7_)I_2_(C_5_H_5_NO)_3_][SnI_3_]·CHCl_3_
*M*_r_	827.77	1319.63
Crystal system, space group	Triclinic, *P* 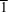	Monoclinic, *C*2/*c*
Temperature (K)	100	200
*a*, *b*, *c* (Å)	8.5224 (3), 9.2972 (4), 16.9400 (8)	40.7321 (18), 8.5279 (4), 20.3457 (9)
α, β, γ (°)	81.649 (1), 75.958 (2), 71.025 (1)	90, 102.553 (2), 90
*V* (Å^3^)	1228.14 (9)	6898.3 (5)
*Z*	2	8
Radiation type	Mo *K*α	Mo *K*α
μ (mm^−1^)	4.83	6.18
Crystal size (mm)	0.33 × 0.18 × 0.12	0.28 × 0.19 × 0.11

Data collection		
Diffractometer	Bruker APEXII CCD	Bruker APEXII CCD
Absorption correction	Multi-scan (*SADABS*; Krause *et al.*, 2015 ▸)	Multi-scan (*SADABS*; Krause *et al.*, 2015 ▸)
*T*_min_, *T*_max_	0.455, 0.693	0.453, 0.712
No. of measured, independent and observed [*I* > 2σ(*I*)] reflections	94618, 5925, 5243	146956, 8327, 6129
*R*_int_	0.088	0.091
(sin θ/λ)_max_ (Å^−1^)	0.661	0.661

Refinement		
*R*[*F*^2^ > 2σ(*F*^2^)], *wR*(*F*^2^), *S*	0.029, 0.069, 1.05	0.032, 0.078, 1.11
No. of reflections	5925	8327
No. of parameters	271	319
No. of restraints	8	0
H-atom treatment	H-atom parameters constrained	H-atom parameters constrained
Δρ_max_, Δρ_min_ (e Å^−3^)	1.43, −1.62	0.94, −1.51

Computer programs: *APEX2* and *SAINT* (Bruker, 2009 ▸), *SHELXS97* (Sheldrick 2008 ▸), *SHELXL2014/7* (Sheldrick, 2015 ▸), *DIAMOND* (Brandenburg, 2006 ▸), *Mercury* (Macrae *et al.* (2020 ▸) and *publCIF*(Westrip, 2010 ▸).

## supplementary crystallographic information

### Diiodido(isopropyl)tris(pyridine N-oxide)tin(IV) iodide (1) Crystal data

**Table:**

[Sn(C_3_H_7_)I_2_(C_5_H_5_NO)_3_]I	*Z* = 2
*M**_r_* = 827.77	*F*(000) = 768
Triclinic, *P*1	*D*_x_ = 2.238 Mg m^−^^3^
*a* = 8.5224 (3) Å	Mo *K*α radiation, λ = 0.71073 Å
*b* = 9.2972 (4) Å	Cell parameters from 9764 reflections
*c* = 16.9400 (8) Å	θ = 2.3–28.6°
α = 81.649 (1)°	µ = 4.83 mm^−^^1^
β = 75.958 (2)°	*T* = 100 K
γ = 71.025 (1)°	Needle, yellow
*V* = 1228.14 (9) Å^3^	0.33 × 0.18 × 0.12 mm

### Diiodido(isopropyl)tris(pyridine N-oxide)tin(IV) iodide (1) Data collection

**Table:**

Bruker APEXII CCD diffractometer	5243 reflections with *I* > 2σ(*I*)
φ and ω scans	*R*_int_ = 0.088
Absorption correction: multi-scan (SADABS; Krause *et al.*, 2015)	θ_max_ = 28.0°, θ_min_ = 2.9°
*T*_min_ = 0.455, *T*_max_ = 0.693	*h* = −11→11
94618 measured reflections	*k* = −12→12
5925 independent reflections	*l* = −22→22

### Diiodido(isopropyl)tris(pyridine N-oxide)tin(IV) iodide (1) Refinement

**Table:**

Refinement on *F*^2^	Hydrogen site location: inferred from neighbouring sites
Least-squares matrix: full	H-atom parameters constrained
*R*[*F*^2^ > 2σ(*F*^2^)] = 0.029	*w* = 1/[σ^2^(*F*_o_^2^) + (0.0184*P*)^2^ + 4.7037*P*] where *P* = (*F*_o_^2^ + 2*F*_c_^2^)/3
*wR*(*F*^2^) = 0.069	(Δ/σ)_max_ = 0.001
*S* = 1.05	Δρ_max_ = 1.43 e Å^−^^3^
5925 reflections	Δρ_min_ = −1.62 e Å^−^^3^
271 parameters	Extinction correction: SHELXL-2014/7 (Sheldrick, 2015), Fc^*^=kFc[1+0.001xFc^2^λ^3^/sin(2θ)]^-1/4^
8 restraints	Extinction coefficient: 0.00180 (15)
Primary atom site location: structure-invariant direct methods	

### Diiodido(isopropyl)tris(pyridine N-oxide)tin(IV) iodide (1) Special details

**Table:**

Geometry. All esds (except the esd in the dihedral angle between two l.s. planes) are estimated using the full covariance matrix. The cell esds are taken into account individually in the estimation of esds in distances, angles and torsion angles; correlations between esds in cell parameters are only used when they are defined by crystal symmetry. An approximate (isotropic) treatment of cell esds is used for estimating esds involving l.s. planes.

### Diiodido(isopropyl)tris(pyridine N-oxide)tin(IV) iodide (1) Fractional atomic coordinates and isotropic or equivalent isotropic displacement parameters (Å^2^)

**Table:**

	*x*	*y*	*z*	*U*_iso_*/*U*_eq_	Occ. (<1)
Sn1	0.33877 (3)	0.84757 (3)	0.82740 (2)	0.01992 (8)	
C1	0.2579 (10)	0.9256 (8)	0.9528 (3)	0.0245 (13)	0.5
H1	0.1578	0.8907	0.9811	0.029*	0.5
C2	0.204 (3)	1.0955 (9)	0.9528 (13)	0.0331 (11)	0.5
H2A	0.3037	1.1317	0.9330	0.050*	0.5
H2B	0.1502	1.1258	1.0084	0.050*	0.5
H2C	0.1233	1.1405	0.9170	0.050*	0.5
C3	0.3958 (11)	0.8534 (13)	0.9993 (6)	0.0331 (11)	0.5
H3A	0.4120	0.7435	1.0086	0.050*	0.5
H3B	0.3643	0.8984	1.0519	0.050*	0.5
H3C	0.5016	0.8710	0.9680	0.050*	0.5
C4	0.3282 (11)	0.9541 (8)	0.9383 (4)	0.0245 (13)	0.5
H4	0.4350	0.9837	0.9253	0.029*	0.5
C5	0.191 (3)	1.1036 (11)	0.9448 (13)	0.0331 (11)	0.5
H5A	0.1941	1.1545	0.9909	0.050*	0.5
H5B	0.0804	1.0861	0.9535	0.050*	0.5
H5C	0.2083	1.1681	0.8944	0.050*	0.5
C6	0.3467 (13)	0.8439 (12)	1.0116 (5)	0.0331 (11)	0.5
H6A	0.4542	0.7622	0.9997	0.050*	0.5
H6B	0.2523	0.8002	1.0256	0.050*	0.5
H6C	0.3456	0.8972	1.0577	0.050*	0.5
I1A	0.00404 (4)	0.91616 (4)	0.81085 (2)	0.03333 (11)	0.9842 (9)
I1B	0.0218 (12)	0.820 (3)	0.8293 (12)	0.03333 (11)	0.0158 (9)
I2A	0.36423 (10)	0.53886 (4)	0.88418 (5)	0.03183 (15)	0.969 (3)
I2B	0.417 (3)	0.5249 (4)	0.8557 (14)	0.03183 (15)	0.031 (3)
O1	0.4447 (3)	0.7754 (3)	0.70685 (16)	0.0227 (6)	
N1	0.3663 (4)	0.7237 (4)	0.66165 (19)	0.0191 (6)	
C11	0.2460 (5)	0.8233 (4)	0.6254 (2)	0.0223 (8)	
H11	0.2143	0.9294	0.6319	0.027*	
C12	0.1693 (5)	0.7683 (5)	0.5784 (2)	0.0269 (9)	
H12	0.0802	0.8363	0.5546	0.032*	
C13	0.2217 (6)	0.6150 (5)	0.5661 (2)	0.0286 (9)	
H13	0.1716	0.5773	0.5325	0.034*	
C14	0.3489 (6)	0.5164 (5)	0.6034 (3)	0.0304 (9)	
H14	0.3869	0.4105	0.5954	0.037*	
C15	0.4186 (5)	0.5730 (4)	0.6516 (3)	0.0260 (8)	
H15	0.5042	0.5062	0.6781	0.031*	
O2	0.6099 (4)	0.8154 (3)	0.81148 (18)	0.0262 (6)	
N2	0.7301 (4)	0.6804 (4)	0.7976 (2)	0.0242 (7)	
C21	0.7980 (5)	0.6384 (5)	0.7211 (3)	0.0274 (9)	
H21	0.7603	0.7032	0.6763	0.033*	
C22	0.9229 (5)	0.5007 (5)	0.7080 (3)	0.0333 (10)	
H22	0.9700	0.4691	0.6541	0.040*	
C23	0.9797 (6)	0.4079 (5)	0.7741 (3)	0.0378 (11)	
H23	1.0656	0.3128	0.7659	0.045*	
C24	0.9097 (6)	0.4562 (5)	0.8511 (3)	0.0405 (11)	
H24	0.9486	0.3953	0.8967	0.049*	
C25	0.7832 (6)	0.5928 (5)	0.8624 (3)	0.0344 (10)	
H25	0.7333	0.6255	0.9159	0.041*	
O3	0.3312 (3)	1.0686 (3)	0.76145 (18)	0.0264 (6)	
N3	0.4763 (4)	1.1026 (3)	0.72691 (19)	0.0201 (6)	
C31	0.5590 (6)	1.0604 (5)	0.6523 (2)	0.0262 (8)	
H31	0.5170	1.0054	0.6236	0.031*	
C32	0.7064 (6)	1.0974 (5)	0.6174 (3)	0.0322 (10)	
H32	0.7669	1.0660	0.5647	0.039*	
C33	0.7661 (5)	1.1788 (5)	0.6581 (3)	0.0324 (10)	
H33	0.8675	1.2043	0.6343	0.039*	
C34	0.6742 (6)	1.2232 (5)	0.7351 (3)	0.0308 (9)	
H34	0.7116	1.2809	0.7645	0.037*	
C35	0.5307 (6)	1.1836 (5)	0.7680 (3)	0.0274 (9)	
H35	0.4682	1.2135	0.8207	0.033*	
I3	0.78826 (3)	0.77318 (3)	0.48262 (2)	0.02366 (8)	

### Diiodido(isopropyl)tris(pyridine N-oxide)tin(IV) iodide (1) Atomic displacement parameters (Å^2^)

**Table:**

	*U*^11^	*U*^22^	*U*^33^	*U*^12^	*U*^13^	*U*^23^
Sn1	0.02376 (14)	0.01856 (13)	0.01909 (14)	−0.00794 (10)	−0.00818 (10)	0.00338 (10)
C1	0.028 (4)	0.024 (3)	0.025 (3)	−0.013 (3)	−0.005 (3)	−0.002 (2)
C2	0.038 (3)	0.0302 (18)	0.021 (2)	−0.0030 (18)	0.0023 (18)	−0.0029 (15)
C3	0.038 (3)	0.0302 (18)	0.021 (2)	−0.0030 (18)	0.0023 (18)	−0.0029 (15)
C4	0.028 (4)	0.024 (3)	0.025 (3)	−0.013 (3)	−0.005 (3)	−0.002 (2)
C5	0.038 (3)	0.0302 (18)	0.021 (2)	−0.0030 (18)	0.0023 (18)	−0.0029 (15)
C6	0.038 (3)	0.0302 (18)	0.021 (2)	−0.0030 (18)	0.0023 (18)	−0.0029 (15)
I1A	0.02310 (15)	0.0481 (2)	0.02945 (16)	−0.01426 (13)	−0.00183 (11)	−0.00302 (14)
I1B	0.02310 (15)	0.0481 (2)	0.02945 (16)	−0.01426 (13)	−0.00183 (11)	−0.00302 (14)
I2A	0.0489 (3)	0.01971 (14)	0.0267 (3)	−0.01293 (14)	−0.0071 (3)	0.00379 (13)
I2B	0.0489 (3)	0.01971 (14)	0.0267 (3)	−0.01293 (14)	−0.0071 (3)	0.00379 (13)
O1	0.0243 (14)	0.0293 (14)	0.0202 (13)	−0.0146 (11)	−0.0071 (11)	−0.0001 (11)
N1	0.0203 (15)	0.0198 (15)	0.0188 (15)	−0.0087 (12)	−0.0050 (12)	0.0006 (12)
C11	0.027 (2)	0.0180 (18)	0.0206 (19)	−0.0080 (15)	−0.0042 (15)	0.0038 (14)
C12	0.028 (2)	0.031 (2)	0.023 (2)	−0.0113 (17)	−0.0086 (16)	0.0071 (16)
C13	0.037 (2)	0.036 (2)	0.020 (2)	−0.0212 (19)	−0.0068 (17)	−0.0008 (17)
C14	0.039 (2)	0.022 (2)	0.032 (2)	−0.0105 (18)	−0.0077 (19)	−0.0044 (17)
C15	0.027 (2)	0.0201 (19)	0.028 (2)	−0.0023 (16)	−0.0093 (17)	0.0002 (16)
O2	0.0270 (14)	0.0179 (13)	0.0367 (16)	−0.0065 (11)	−0.0143 (12)	0.0005 (11)
N2	0.0225 (16)	0.0177 (15)	0.0359 (19)	−0.0063 (13)	−0.0145 (14)	0.0019 (14)
C21	0.0217 (19)	0.028 (2)	0.035 (2)	−0.0123 (16)	−0.0064 (17)	0.0020 (17)
C22	0.023 (2)	0.033 (2)	0.047 (3)	−0.0152 (18)	−0.0028 (19)	−0.007 (2)
C23	0.023 (2)	0.021 (2)	0.068 (3)	−0.0055 (17)	−0.011 (2)	0.000 (2)
C24	0.039 (3)	0.030 (2)	0.052 (3)	−0.005 (2)	−0.023 (2)	0.011 (2)
C25	0.038 (2)	0.028 (2)	0.038 (3)	−0.0046 (19)	−0.018 (2)	0.0033 (19)
O3	0.0206 (13)	0.0195 (13)	0.0354 (16)	−0.0069 (11)	−0.0037 (12)	0.0081 (12)
N3	0.0195 (15)	0.0138 (14)	0.0238 (16)	−0.0038 (12)	−0.0030 (13)	0.0030 (12)
C31	0.037 (2)	0.024 (2)	0.0208 (19)	−0.0147 (17)	−0.0071 (17)	0.0021 (15)
C32	0.038 (2)	0.024 (2)	0.028 (2)	−0.0114 (18)	0.0057 (18)	0.0004 (17)
C33	0.024 (2)	0.024 (2)	0.044 (3)	−0.0093 (17)	0.0008 (19)	0.0056 (18)
C34	0.032 (2)	0.029 (2)	0.037 (2)	−0.0161 (18)	−0.0102 (19)	0.0009 (18)
C35	0.035 (2)	0.024 (2)	0.025 (2)	−0.0127 (17)	−0.0036 (17)	−0.0012 (16)
I3	0.02654 (14)	0.01875 (13)	0.02718 (14)	−0.00621 (10)	−0.00905 (10)	−0.00196 (10)

### Diiodido(isopropyl)tris(pyridine N-oxide)tin(IV) iodide (1) Geometric parameters (Å, º)

**Table:**

Sn1—O1	2.132 (3)	C11—H11	0.9500
Sn1—O3	2.181 (3)	C12—C13	1.379 (6)
Sn1—O2	2.184 (3)	C12—H12	0.9500
Sn1—C4	2.219 (5)	C13—C14	1.390 (6)
Sn1—C1	2.219 (5)	C13—H13	0.9500
Sn1—I1B	2.7883 (10)	C14—C15	1.364 (6)
Sn1—I1A	2.7886 (4)	C14—H14	0.9500
Sn1—I2A	2.8468 (4)	C15—H15	0.9500
Sn1—I2B	2.8476 (10)	O2—N2	1.347 (4)
C1—C3	1.495 (5)	N2—C21	1.342 (6)
C1—C2	1.496 (5)	N2—C25	1.348 (5)
C1—H1	1.0000	C21—C22	1.380 (6)
C2—H2A	0.9800	C21—H21	0.9500
C2—H2B	0.9800	C22—C23	1.394 (7)
C2—H2C	0.9800	C22—H22	0.9500
C3—H3A	0.9800	C23—C24	1.371 (7)
C3—H3B	0.9800	C23—H23	0.9500
C3—H3C	0.9800	C24—C25	1.376 (6)
C4—C6	1.495 (5)	C24—H24	0.9500
C4—C5	1.495 (5)	C25—H25	0.9500
C4—H4	1.0000	O3—N3	1.349 (4)
C5—H5A	0.9800	N3—C31	1.334 (5)
C5—H5B	0.9800	N3—C35	1.343 (5)
C5—H5C	0.9800	C31—C32	1.381 (6)
C6—H6A	0.9800	C31—H31	0.9500
C6—H6B	0.9800	C32—C33	1.371 (7)
C6—H6C	0.9800	C32—H32	0.9500
I1A—I1B	0.88 (2)	C33—C34	1.392 (6)
I2A—I2B	0.57 (3)	C33—H33	0.9500
O1—N1	1.352 (4)	C34—C35	1.358 (6)
N1—C11	1.345 (5)	C34—H34	0.9500
N1—C15	1.348 (5)	C35—H35	0.9500
C11—C12	1.381 (6)		
			
O1—Sn1—O3	80.74 (11)	C4—C6—H6B	109.5
O1—Sn1—O2	75.61 (11)	H6A—C6—H6B	109.5
O3—Sn1—O2	84.77 (10)	C4—C6—H6C	109.5
O1—Sn1—C4	155.2 (2)	H6A—C6—H6C	109.5
O3—Sn1—C4	87.0 (2)	H6B—C6—H6C	109.5
O2—Sn1—C4	81.9 (2)	I1B—I1A—Sn1	80.9 (3)
O1—Sn1—C1	173.3 (2)	I1A—I1B—Sn1	80.9 (3)
O3—Sn1—C1	97.6 (2)	I2B—I2A—Sn1	84.3 (3)
O2—Sn1—C1	97.8 (2)	I2A—I2B—Sn1	84.2 (3)
O1—Sn1—I1B	93.8 (4)	N1—O1—Sn1	125.3 (2)
O3—Sn1—I1B	103.4 (5)	C11—N1—C15	122.4 (3)
O2—Sn1—I1B	165.6 (5)	C11—N1—O1	119.7 (3)
C4—Sn1—I1B	110.0 (5)	C15—N1—O1	117.9 (3)
C1—Sn1—I1B	92.9 (4)	N1—C11—C12	118.7 (4)
O1—Sn1—I1A	94.17 (7)	N1—C11—H11	120.6
O3—Sn1—I1A	85.50 (7)	C12—C11—H11	120.6
O2—Sn1—I1A	166.89 (8)	C13—C12—C11	120.3 (4)
C4—Sn1—I1A	106.3 (2)	C13—C12—H12	119.9
C1—Sn1—I1A	92.2 (2)	C11—C12—H12	119.9
I1B—Sn1—I1A	18.2 (5)	C12—C13—C14	119.1 (4)
O1—Sn1—I2A	88.79 (8)	C12—C13—H13	120.4
O3—Sn1—I2A	169.37 (8)	C14—C13—H13	120.4
O2—Sn1—I2A	94.43 (7)	C15—C14—C13	119.4 (4)
C4—Sn1—I2A	103.4 (2)	C15—C14—H14	120.3
C1—Sn1—I2A	93.0 (2)	C13—C14—H14	120.3
I1B—Sn1—I2A	75.3 (5)	N1—C15—C14	120.1 (4)
I1A—Sn1—I2A	93.510 (16)	N1—C15—H15	120.0
O1—Sn1—I2B	78.3 (5)	C14—C15—H15	120.0
O3—Sn1—I2B	158.8 (5)	N2—O2—Sn1	123.5 (2)
O2—Sn1—I2B	87.1 (4)	C21—N2—O2	120.4 (3)
C4—Sn1—I2B	111.2 (4)	C21—N2—C25	121.8 (4)
C1—Sn1—I2B	102.8 (5)	O2—N2—C25	117.8 (4)
I1B—Sn1—I2B	81.1 (6)	N2—C21—C22	119.6 (4)
I1A—Sn1—I2B	99.0 (3)	N2—C21—H21	120.2
I2A—Sn1—I2B	11.5 (5)	C22—C21—H21	120.2
C3—C1—C2	111.0 (10)	C21—C22—C23	119.7 (5)
C3—C1—Sn1	110.4 (5)	C21—C22—H22	120.1
C2—C1—Sn1	112.2 (9)	C23—C22—H22	120.1
C3—C1—H1	107.7	C24—C23—C22	118.9 (4)
C2—C1—H1	107.7	C24—C23—H23	120.5
Sn1—C1—H1	107.7	C22—C23—H23	120.5
C1—C2—H2A	109.5	C23—C24—C25	120.0 (4)
C1—C2—H2B	109.5	C23—C24—H24	120.0
H2A—C2—H2B	109.5	C25—C24—H24	120.0
C1—C2—H2C	109.5	N2—C25—C24	119.9 (5)
H2A—C2—H2C	109.5	N2—C25—H25	120.0
H2B—C2—H2C	109.5	C24—C25—H25	120.0
C1—C3—H3A	109.5	N3—O3—Sn1	120.4 (2)
C1—C3—H3B	109.5	C31—N3—C35	121.5 (3)
H3A—C3—H3B	109.5	C31—N3—O3	119.9 (3)
C1—C3—H3C	109.5	C35—N3—O3	118.5 (3)
H3A—C3—H3C	109.5	N3—C31—C32	119.3 (4)
H3B—C3—H3C	109.5	N3—C31—H31	120.4
C6—C4—C5	120.2 (11)	C32—C31—H31	120.4
C6—C4—Sn1	113.6 (6)	C33—C32—C31	120.6 (4)
C5—C4—Sn1	109.9 (8)	C33—C32—H32	119.7
C6—C4—H4	103.7	C31—C32—H32	119.7
C5—C4—H4	103.7	C32—C33—C34	118.2 (4)
Sn1—C4—H4	103.7	C32—C33—H33	120.9
C4—C5—H5A	109.5	C34—C33—H33	120.9
C4—C5—H5B	109.5	C35—C34—C33	119.6 (4)
H5A—C5—H5B	109.5	C35—C34—H34	120.2
C4—C5—H5C	109.5	C33—C34—H34	120.2
H5A—C5—H5C	109.5	N3—C35—C34	120.7 (4)
H5B—C5—H5C	109.5	N3—C35—H35	119.7
C4—C6—H6A	109.5	C34—C35—H35	119.7

### Diiodido(isopropyl)tris(pyridine N-oxide)tin(IV) triiodidostannate(II) deuterochloroform monosolvate (2) Crystal data

**Table:**

[Sn(C_3_H_7_)I_2_(C_5_H_5_NO)_3_][SnI_3_]·CHCl_3_	*F*(000) = 4784
*M**_r_* = 1319.63	*D*_x_ = 2.541 Mg m^−^^3^
Monoclinic, *C*2/*c*	Mo *K*α radiation, λ = 0.71073 Å
*a* = 40.7321 (18) Å	Cell parameters from 9880 reflections
*b* = 8.5279 (4) Å	θ = 2.6–26.1°
*c* = 20.3457 (9) Å	µ = 6.18 mm^−^^1^
β = 102.553 (2)°	*T* = 200 K
*V* = 6898.3 (5) Å^3^	Plate, yellow
*Z* = 8	0.28 × 0.19 × 0.11 mm

### Diiodido(isopropyl)tris(pyridine N-oxide)tin(IV) triiodidostannate(II) deuterochloroform monosolvate (2) Data collection

**Table:**

Bruker APEXII CCD diffractometer	6129 reflections with *I* > 2σ(*I*)
φ and ω scans	*R*_int_ = 0.091
Absorption correction: multi-scan (SADABS; Krause *et al.*, 2015)	θ_max_ = 28.0°, θ_min_ = 2.4°
*T*_min_ = 0.453, *T*_max_ = 0.712	*h* = −53→53
146956 measured reflections	*k* = −11→11
8327 independent reflections	*l* = −26→26

### Diiodido(isopropyl)tris(pyridine N-oxide)tin(IV) triiodidostannate(II) deuterochloroform monosolvate (2) Refinement

**Table:**

Refinement on *F*^2^	Hydrogen site location: inferred from neighbouring sites
Least-squares matrix: full	H-atom parameters constrained
*R*[*F*^2^ > 2σ(*F*^2^)] = 0.032	*w* = 1/[σ^2^(*F*_o_^2^) + (0.0189*P*)^2^ + 43.8049*P*] where *P* = (*F*_o_^2^ + 2*F*_c_^2^)/3
*wR*(*F*^2^) = 0.078	(Δ/σ)_max_ = 0.001
*S* = 1.11	Δρ_max_ = 0.94 e Å^−^^3^
8327 reflections	Δρ_min_ = −1.51 e Å^−^^3^
319 parameters	Extinction correction: SHELXL-2014/7 (Sheldrick 2015), Fc^*^=kFc[1+0.001xFc^2^λ^3^/sin(2θ)]^-1/4^
0 restraints	Extinction coefficient: 0.000136 (8)
Primary atom site location: structure-invariant direct methods	

### Diiodido(isopropyl)tris(pyridine N-oxide)tin(IV) triiodidostannate(II) deuterochloroform monosolvate (2) Special details

**Table:**

Geometry. All esds (except the esd in the dihedral angle between two l.s. planes) are estimated using the full covariance matrix. The cell esds are taken into account individually in the estimation of esds in distances, angles and torsion angles; correlations between esds in cell parameters are only used when they are defined by crystal symmetry. An approximate (isotropic) treatment of cell esds is used for estimating esds involving l.s. planes.

### Diiodido(isopropyl)tris(pyridine N-oxide)tin(IV) triiodidostannate(II) deuterochloroform monosolvate (2) Fractional atomic coordinates and isotropic or equivalent isotropic displacement parameters (Å^2^)

**Table:**

	*x*	*y*	*z*	*U*_iso_*/*U*_eq_	
Sn1	0.07997 (2)	0.30880 (5)	0.45557 (2)	0.04285 (11)	
C1	0.02477 (16)	0.3347 (12)	0.4161 (3)	0.077 (2)	
H1	0.0205	0.4497	0.4186	0.093*	
C2	0.0146 (2)	0.2971 (14)	0.3464 (4)	0.112 (4)	
H2A	0.0195	0.1865	0.3395	0.134*	
H2B	0.0268	0.3632	0.3204	0.134*	
H2C	−0.0097	0.3157	0.3312	0.134*	
C3	0.00585 (17)	0.2631 (15)	0.4621 (4)	0.118 (4)	
H3A	−0.0180	0.2567	0.4398	0.176*	
H3B	0.0084	0.3274	0.5028	0.176*	
H3C	0.0146	0.1575	0.4743	0.176*	
I12	0.10079 (2)	0.26591 (7)	0.33365 (2)	0.06953 (15)	
I11	0.08419 (2)	−0.00522 (5)	0.50013 (2)	0.05628 (12)	
O1	0.13196 (8)	0.3397 (4)	0.50774 (17)	0.0403 (8)	
N1	0.15855 (10)	0.2490 (6)	0.5026 (2)	0.0396 (10)	
C11	0.17589 (13)	0.2835 (8)	0.4550 (3)	0.0486 (14)	
H11	0.1688	0.3668	0.4241	0.058*	
C12	0.20410 (14)	0.1962 (8)	0.4517 (3)	0.0579 (17)	
H12	0.2159	0.2158	0.4172	0.069*	
C13	0.21485 (13)	0.0818 (8)	0.4982 (3)	0.0545 (16)	
H13	0.2345	0.0229	0.4967	0.065*	
C14	0.19708 (13)	0.0516 (8)	0.5478 (3)	0.0493 (14)	
H14	0.2043	−0.0278	0.5805	0.059*	
C15	0.16877 (12)	0.1388 (7)	0.5486 (2)	0.0423 (13)	
H15	0.1564	0.1201	0.5824	0.051*	
O2	0.08545 (9)	0.5609 (5)	0.45361 (19)	0.0492 (10)	
N2	0.10945 (12)	0.6393 (6)	0.4303 (2)	0.0472 (12)	
C21	0.10139 (18)	0.7087 (9)	0.3698 (3)	0.0632 (19)	
H21	0.0794	0.6961	0.3426	0.076*	
C22	0.1243 (2)	0.7966 (9)	0.3471 (3)	0.077 (2)	
H22	0.1183	0.8448	0.3041	0.092*	
C23	0.1561 (2)	0.8166 (9)	0.3859 (4)	0.074 (2)	
H23	0.1723	0.8772	0.3700	0.089*	
C24	0.16394 (17)	0.7461 (8)	0.4488 (3)	0.0599 (17)	
H24	0.1858	0.7577	0.4767	0.072*	
C25	0.13996 (14)	0.6597 (7)	0.4702 (3)	0.0486 (14)	
H25	0.1450	0.6138	0.5138	0.058*	
O3	0.07154 (8)	0.3721 (5)	0.55466 (17)	0.0473 (10)	
N3	0.09476 (10)	0.4454 (6)	0.6019 (2)	0.0392 (10)	
C31	0.11610 (12)	0.3596 (8)	0.6463 (2)	0.0434 (13)	
H31	0.1153	0.2485	0.6436	0.052*	
C32	0.13918 (13)	0.4318 (8)	0.6960 (3)	0.0496 (15)	
H32	0.1544	0.3707	0.7280	0.060*	
C33	0.14039 (14)	0.5927 (8)	0.6997 (3)	0.0517 (15)	
H33	0.1565	0.6440	0.7337	0.062*	
C34	0.11792 (15)	0.6778 (8)	0.6534 (3)	0.0496 (14)	
H34	0.1184	0.7891	0.6552	0.060*	
C35	0.09471 (14)	0.6026 (8)	0.6043 (3)	0.0473 (14)	
H35	0.0789	0.6612	0.5725	0.057*	
Sn2	0.24796 (2)	0.79515 (5)	0.25634 (2)	0.04113 (10)	
I21	0.21911 (2)	0.55232 (5)	0.33816 (2)	0.04399 (10)	
I22	0.18709 (2)	1.00095 (5)	0.23342 (2)	0.04525 (10)	
I23	0.27972 (2)	0.97219 (6)	0.37615 (2)	0.05848 (13)	
C4	0.03371 (16)	0.1989 (9)	0.6593 (4)	0.0677 (19)	
H4	0.0430	0.2117	0.6180	0.081*	
Cl1	−0.00929 (5)	0.1675 (3)	0.63373 (14)	0.1039 (8)	
Cl2	0.04250 (5)	0.3705 (3)	0.70730 (10)	0.0815 (6)	
Cl3	0.05415 (5)	0.0359 (3)	0.70536 (12)	0.0887 (6)	

### Diiodido(isopropyl)tris(pyridine N-oxide)tin(IV) triiodidostannate(II) deuterochloroform monosolvate (2) Atomic displacement parameters (Å^2^)

**Table:**

	*U*^11^	*U*^22^	*U*^33^	*U*^12^	*U*^13^	*U*^23^
Sn1	0.02725 (17)	0.0714 (3)	0.02831 (18)	0.00606 (17)	0.00268 (13)	−0.00730 (17)
C1	0.040 (3)	0.129 (7)	0.055 (4)	0.005 (4)	−0.007 (3)	−0.014 (4)
C2	0.062 (5)	0.165 (10)	0.092 (6)	−0.008 (6)	−0.017 (5)	0.028 (7)
C3	0.026 (3)	0.233 (13)	0.089 (6)	0.009 (5)	0.000 (4)	−0.018 (7)
I12	0.0773 (3)	0.1009 (4)	0.0335 (2)	0.0170 (3)	0.0188 (2)	−0.0052 (2)
I11	0.0451 (2)	0.0678 (3)	0.0562 (2)	−0.00668 (19)	0.01153 (18)	−0.0076 (2)
O1	0.0242 (17)	0.057 (2)	0.0388 (19)	0.0067 (16)	0.0060 (14)	−0.0019 (17)
N1	0.023 (2)	0.061 (3)	0.034 (2)	0.0021 (19)	0.0050 (17)	0.002 (2)
C11	0.037 (3)	0.071 (4)	0.041 (3)	0.005 (3)	0.016 (2)	0.010 (3)
C12	0.034 (3)	0.088 (5)	0.057 (4)	0.009 (3)	0.022 (3)	0.017 (3)
C13	0.028 (3)	0.081 (5)	0.056 (4)	0.014 (3)	0.013 (3)	0.008 (3)
C14	0.031 (3)	0.073 (4)	0.041 (3)	0.007 (3)	0.002 (2)	0.008 (3)
C15	0.025 (2)	0.072 (4)	0.030 (3)	−0.001 (2)	0.004 (2)	0.005 (3)
O2	0.036 (2)	0.070 (3)	0.043 (2)	0.0123 (19)	0.0114 (17)	−0.001 (2)
N2	0.051 (3)	0.062 (3)	0.031 (2)	0.017 (2)	0.012 (2)	0.006 (2)
C21	0.074 (4)	0.083 (5)	0.032 (3)	0.029 (4)	0.009 (3)	0.006 (3)
C22	0.116 (7)	0.082 (5)	0.040 (4)	0.029 (5)	0.034 (4)	0.019 (4)
C23	0.084 (5)	0.080 (5)	0.072 (5)	0.016 (4)	0.050 (4)	0.008 (4)
C24	0.057 (4)	0.065 (4)	0.063 (4)	0.010 (3)	0.026 (3)	0.005 (3)
C25	0.046 (3)	0.063 (4)	0.039 (3)	0.010 (3)	0.013 (3)	0.004 (3)
O3	0.0303 (18)	0.081 (3)	0.0298 (18)	−0.0082 (19)	0.0046 (15)	−0.0142 (18)
N3	0.026 (2)	0.064 (3)	0.028 (2)	−0.001 (2)	0.0044 (17)	−0.005 (2)
C31	0.032 (3)	0.066 (4)	0.033 (3)	0.000 (3)	0.009 (2)	−0.003 (3)
C32	0.030 (3)	0.082 (5)	0.034 (3)	−0.006 (3)	0.002 (2)	0.001 (3)
C33	0.041 (3)	0.078 (5)	0.037 (3)	−0.012 (3)	0.010 (2)	−0.011 (3)
C34	0.051 (3)	0.061 (4)	0.038 (3)	−0.006 (3)	0.011 (3)	−0.007 (3)
C35	0.037 (3)	0.069 (4)	0.038 (3)	0.005 (3)	0.011 (2)	−0.004 (3)
Sn2	0.03211 (18)	0.0585 (2)	0.03364 (19)	0.00321 (17)	0.00892 (14)	0.00114 (17)
I21	0.03067 (17)	0.0658 (3)	0.03681 (18)	0.00703 (16)	0.01019 (14)	0.01054 (17)
I22	0.03276 (18)	0.0618 (2)	0.04178 (19)	0.00575 (16)	0.00928 (14)	0.00537 (17)
I23	0.0412 (2)	0.0936 (3)	0.0390 (2)	−0.0066 (2)	0.00527 (16)	−0.0089 (2)
C4	0.049 (4)	0.081 (5)	0.074 (5)	−0.003 (3)	0.014 (3)	0.002 (4)
Cl1	0.0492 (10)	0.1056 (17)	0.150 (2)	−0.0147 (11)	0.0054 (12)	0.0017 (16)
Cl2	0.0779 (13)	0.0957 (15)	0.0771 (12)	−0.0138 (11)	0.0305 (10)	−0.0118 (11)
Cl3	0.0740 (13)	0.0922 (15)	0.0928 (15)	0.0099 (11)	0.0026 (11)	0.0101 (12)

### Diiodido(isopropyl)tris(pyridine N-oxide)tin(IV) triiodidostannate(II) deuterochloroform monosolvate (2) Geometric parameters (Å, º)

**Table:**

Sn1—O2	2.163 (4)	C24—C25	1.368 (9)
Sn1—O1	2.169 (3)	O3—N3	1.347 (5)
Sn1—O3	2.185 (3)	N3—C31	1.329 (7)
Sn1—C1	2.228 (6)	N3—C35	1.341 (8)
Sn1—I12	2.8144 (5)	C31—C32	1.368 (7)
Sn1—I11	2.8206 (6)	C32—C33	1.375 (9)
C1—C2	1.426 (11)	C33—C34	1.370 (8)
C1—C3	1.467 (12)	C34—C35	1.376 (8)
O1—N1	1.354 (5)	Sn2—I23	2.9196 (6)
N1—C15	1.328 (7)	Sn2—I22	2.9903 (5)
N1—C11	1.349 (6)	Sn2—I21	3.0481 (5)
C11—C12	1.383 (8)	Sn2—I21^i^	3.3802 (5)
C12—C13	1.364 (9)	Sn2—I22^ii^	3.6188 (5)
C13—C14	1.387 (8)	Sn2—I23^ii^	3.8473 (6)
C14—C15	1.376 (8)	I21—Sn2^ii^	3.3802 (5)
O2—N2	1.353 (6)	I22—Sn2^i^	3.6188 (5)
N2—C25	1.340 (7)	I23—Sn2^i^	3.8473 (6)
N2—C21	1.341 (7)	C4—Cl1	1.736 (7)
C21—C22	1.353 (11)	C4—Cl2	1.753 (8)
C22—C23	1.372 (11)	C4—Cl3	1.779 (8)
C23—C24	1.387 (10)		
			
O2—Sn1—O1	78.14 (14)	C22—C23—C24	118.4 (7)
O2—Sn1—O3	78.98 (15)	C25—C24—C23	119.3 (7)
O1—Sn1—O3	81.42 (12)	N2—C25—C24	120.6 (6)
O2—Sn1—C1	89.7 (3)	N3—O3—Sn1	123.1 (3)
O1—Sn1—C1	165.0 (2)	C31—N3—C35	122.1 (5)
O3—Sn1—C1	87.7 (2)	C31—N3—O3	119.0 (5)
O2—Sn1—I12	93.49 (10)	C35—N3—O3	118.9 (4)
O1—Sn1—I12	89.88 (9)	N3—C31—C32	119.9 (6)
O3—Sn1—I12	169.47 (10)	C31—C32—C33	120.0 (6)
C1—Sn1—I12	99.74 (18)	C34—C33—C32	118.7 (5)
O2—Sn1—I11	161.88 (10)	C33—C34—C35	120.2 (6)
O1—Sn1—I11	88.47 (10)	N3—C35—C34	119.1 (6)
O3—Sn1—I11	87.07 (12)	I23—Sn2—I22	91.639 (16)
C1—Sn1—I11	101.3 (3)	I23—Sn2—I21	92.798 (16)
I12—Sn1—I11	98.680 (19)	I22—Sn2—I21	94.528 (14)
C2—C1—C3	117.4 (8)	I23—Sn2—I21^i^	88.901 (15)
C2—C1—Sn1	113.0 (6)	I22—Sn2—I21^i^	87.046 (14)
C3—C1—Sn1	111.0 (5)	I21—Sn2—I21^i^	177.647 (18)
N1—O1—Sn1	127.3 (3)	I23—Sn2—I22^ii^	97.168 (15)
C15—N1—C11	121.8 (5)	I22—Sn2—I22^ii^	170.674 (17)
C15—N1—O1	118.9 (4)	I21—Sn2—I22^ii^	82.027 (13)
C11—N1—O1	118.9 (4)	I21^i^—Sn2—I22^ii^	96.151 (12)
N1—C11—C12	119.2 (5)	I23—Sn2—I23^ii^	164.661 (19)
C13—C12—C11	119.8 (5)	I22—Sn2—I23^ii^	101.773 (14)
C12—C13—C14	119.9 (5)	I21—Sn2—I23^ii^	78.886 (13)
C15—C14—C13	118.7 (5)	I21^i^—Sn2—I23^ii^	99.090 (12)
N1—C15—C14	120.6 (5)	I22^ii^—Sn2—I23^ii^	69.102 (11)
N2—O2—Sn1	125.7 (3)	Sn2—I21—Sn2^ii^	83.248 (9)
C25—N2—C21	120.6 (6)	Sn2—I22—Sn2^i^	80.039 (9)
C25—N2—O2	119.9 (4)	Sn2—I23—Sn2^i^	77.057 (10)
C21—N2—O2	119.2 (5)	Cl1—C4—Cl2	111.5 (4)
N2—C21—C22	120.5 (7)	Cl1—C4—Cl3	111.3 (4)
C21—C22—C23	120.5 (6)	Cl2—C4—Cl3	110.1 (4)

Symmetry codes: (i) −*x*+1/2, *y*+1/2, −*z*+1/2; (ii) −*x*+1/2, *y*−1/2, −*z*+1/2.
